# Multi-scale Data Improves Performance of Machine Learning Model for Long COVID Prediction

**DOI:** 10.21203/rs.3.rs-7234976/v1

**Published:** 2025-08-31

**Authors:** Wei-Qi Wei, Christopher Guardo, Xinmeng Zhang, Srushti Gandireddy, Chao Yan, Vern Kerchberger, Alyson Dickson, Emily Pfaff, Hiral Master, Melissa Basford, Christopher Chute, Nguyen Tran, Salvatore Manusco, Toufeeq Syed, Zhongming Zhao, QiPing Feng, Melissa Haendel, Christopher Lunt, Paul Harris, Lang Li, Geoffrey Ginsburg, Joshua Denny, Dan Roden

**Affiliations:** Vanderbilt University Medical Center; Vanderbilt University Medical Center; Vanderbilt University Medical Center; Vanderbilt University Medical Center; Vanderbilt University Medical Center; Vanderbilt University Medical Center; Vanderbilt University Medical Center; University of North Carolina, USA; Vanderbilt University Medical; Vanderbilt Institute of Clinical and Translational Research/Vanderbilt University Medical Center; Johns Hopkins University; Stanford University School of Medicine; Oakland University; UTHealth Houston; UTHealth Houston; Vanderbilt University Medical Center; University of Colorado Anschutz Medical Campus; All of Us Research Program; Vanderbilt University Medical Center; Department of Biomedical Informatics, the Ohio State University; All of US Research Program National Institutes of Health; Vanderbilt University Medical Center; Vanderbilt University Medical Center, Nashville, TN

## Abstract

Long COVID affects a substantial proportion of the over 778 million individuals infected with SARS-CoV-2, yet predictive models remain limited in scope. While existing efforts, such as the National COVID Cohort Collaborative (N3C), have leveraged electronic health record (EHR) data for risk prediction, accumulating evidence points to additional contributions from social, behavioral, and genetic factors. Using a diverse cohort of SARS-CoV-2-infected individuals (n>17,200) from the NIH All of Us Research Program, we investigated whether integrating EHR data with survey-based and genomic information improves model performance. Our multi-scale approach outperformed EHR-only models original AUROC 0.736 (95% CI: 0.730, 0.741), achieving an AUROC of 0.748 (0.741,0.755). Among the top predictors, active-duty service status, self-reported fatigue, and chr19:4719431:G:A_A were among the most informative survey and genetic features. These findings highlight the importance of incorporating multi-scale data to improve risk stratification and inform personalized interventions for long COVID.

## INTRODUCTION

Post-acute sequelae of SARS-CoV-2 infection, referred to as long COVID,^1^ has emerged as a growing global public health crisis. With over 769 million confirmed COVID-19 cases worldwide, it is estimated that more than 400 million individuals^2^ have experienced or are at risk for persistent symptoms following initial infections. Despite its wide-ranging clinical, psychological, and social impacts, long COVID remains poorly understood and understudied, posing a major challenge to public health systems.^3^

One of the enduring challenges in long COVID research is accurate prediction of who will develop the condition. To address this, the National COVID Cohort Collaborative (N3C)^4^ assembled a large electronic health record (EHR) repository of SARS-CoV-2-infected individuals across the United States, developed a machine learning (ML)-based model to predict long COVID cases, and validated it using data from the NIH All of Us Research Program (AoU).^5,6 7^ While this model marked a critical advancement, it was trained exclusively on structured EHR data—reflecting only what is captured during clinical encounters—which may limit its capability and predictive accuracy.

Since the initial development of the N3C model in 2022^6^, research has increasingly pointed to additional determinants of long COVID beyond clinical records. Genome-wide association studies have identified multiple genetic variants associated with COVID-19 severity, including a robust association between the Forkhead Box P4 gene (*FOXP4*) and long COVID risk. More than 20 variants have been linked to COVID-19 acquisition and hospitalization, suggesting that genetic architecture may also contribute to persistent post-viral symptoms.^8^

In addition, social and behavioral factors—often captured through surveys—have been shown to influence both acute and long-term COVID-19 outcomes. Findings from AoU, for example, revealed significant reductions in physical activity (e.g., step count) following the pandemic, with disproportionate impacts on socioeconomically disadvantaged populations^9^. Further research indicates that employment status affects mobility and self-care ability, while living with others is generally associated with more favorable outcomes for individuals with long COVID.^10^ Other studies have further linked smoking, employment status, financial hardship, and anxiety and depressive symptomatology to long COVID risk and recovery, underscoring the critical role of social determinants of health (SDOH).^11,12^

Recognizing the multifactorial nature of long COVID, we hypothesize that integrating EHR data with survey-based SDOH and genetic information can enhance risk prediction. In this study, we leverage the richly phenotyped and genomically profiled AoU cohort to develop and evaluate a multi-scale predictive model for identifying individuals with high long COVID risk, aiming to go beyond the traditional EHR-based approach.

## RESULTS

### Study population

**Figure 1:**
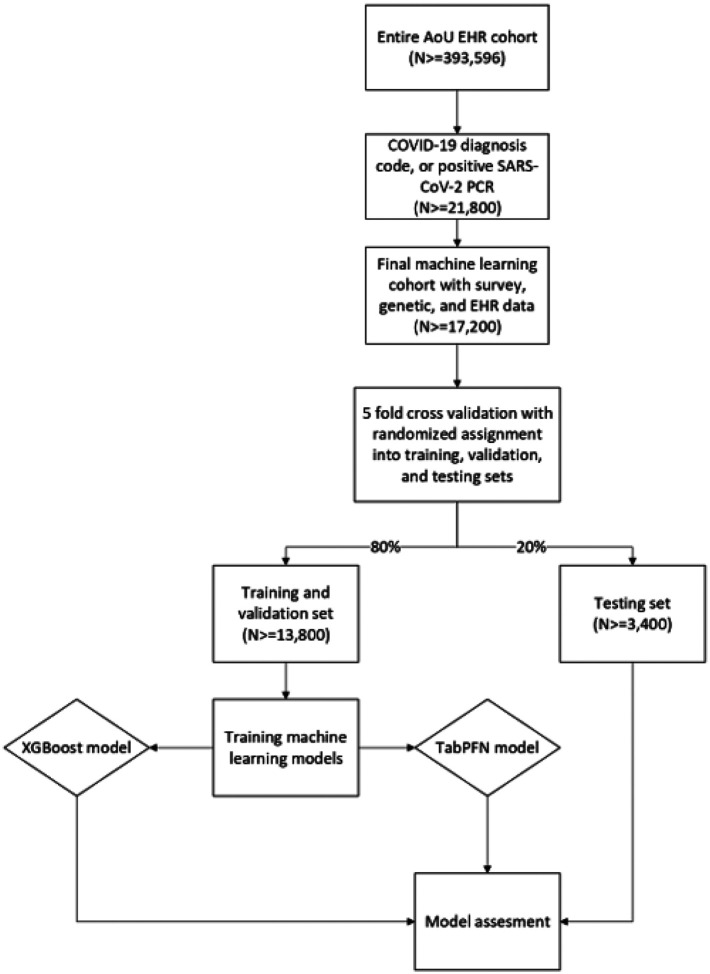
Cohort generation and training, testing, and validation set assignment.

We constructed our study cohort using criteria consistent with those applied in the development of the original N3C algorithm for long COVID prediction^6^. First, we limited the AOU participant pool to those with a documented COVID19 diagnosis in their EHRs. Second, we further restricted this subset to participants with data available from EHR, survey, and genomic data to ensure data quality and support our multi-scale modeling approach. Additionally, to be consistent with the definition of long COVID introduced by Centers for Disease Control and Prevention (CDC) of the United States, we limited the scope of each participant’s data to a defined post-infection window^13^. Participants were labeled as having COVID-19 if a participant had either of the following inclusion criteria: 1) an International Classification of Diseases, Tenth Revision, Clinical Modification (ICD-10-CM) COVID-19 diagnosis code (i.e., U07.1) from an inpatient or emergency department visit, or 2) a positive SARS-CoV-2 PCR or antigen test. Details of the cohort construction are provided in the Methods (Figure 1).

Our final analytical cohort consisted of 17,256 AoU participants, of whom 652 (3.78%) had the diagnosis ICD 10 code U09, identifying them as long COVID cases. The All of Us Research Program defines race, ethnicity, and gender as self-reported variables from a participant-level survey. Further, ethnicity and race are reported together in this survey. In our final cohort, 11,460 participants identified as women, and 5539 participants identified as men. The race and ethnicity distribution in our final cohort is as follows: 3032 participants self-identified as Hispanic or Latino, 8938 participants identified as white, 3222 participants identified as Black, and 1101 participants reported more than one race, and were categorized separately. The exact number of participants in minority populations of size less than 20 are hidden following All Of Us terms of service. The full demographic distribution of the study cohort is shown in Table 1.

### Machine Learning Methods

We employed two machine learning approaches to predict long COVID: XGBoost^14^ and TabPFN^15^. XGBoost, a gradient-boosted decision tree algorithm, was selected due to its widespread use in predictive modeling as well as its application in the original N3C long COVID prediction model.^6^ We also implemented TabPFN—an attention-based transformer model optimized for tabular data. As a state-of-the-art approach, recent studies have shown that TabPFN can outperform tree-based models (e.g., LightGBM and XGBoost) when trained and evaluated on the same datasets. Given this evidence, we compared TabPFN to XGBoost in our long COVID prediction task. To reduce bias in performance evaluation, we randomly split the final cohort data into two portions: one for training the model, and the other for evaluating it. We did this 10 times independently, i.e., a dataset for model testing and the other for training and validation, where a 5-fold cross-validation was performed.

### Model Results

The AUROC of the XGBoost Model using EHR data alone was 0.736 with a 95% confidence interval (CI) of (0.730, 0.741) in the current version of AoU data (v8). This result is comparable to the N3C model’s original performance, which had a reported AUROC of 0.721 in a study that used the previous version of AoU (v7)^7^. This difference may be due to the larger number of participants and longer time span of data in the newest update of AoU data in version 8.

**Figure 2:**
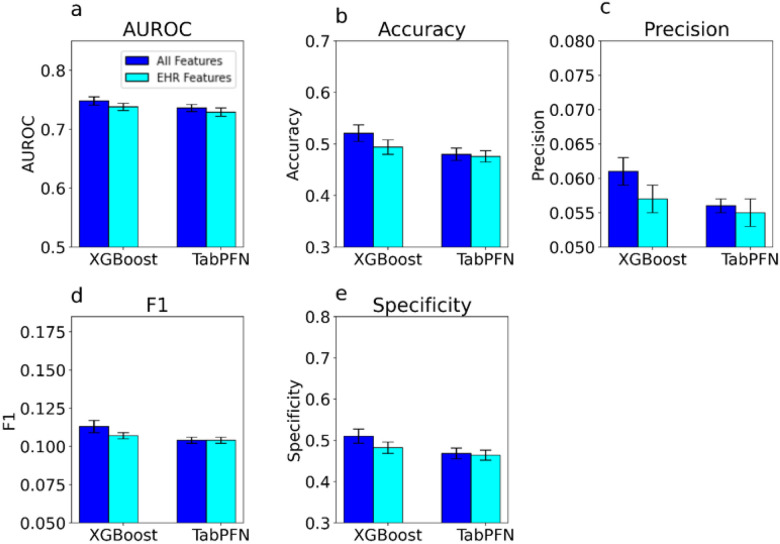
Average AUROC, Accuracy, Precision, F1, and Specificity of Models

The AUROC improved on average when survey and genetic data was added to the algorithm’s predictive method. The addition of survey and genetic data features to the XGBoost model modestly improved AUROC from 0.736 (0.730, 0.741) to a mean (95% CI) of 0.748 (0.741, 0.755). We performed a Wilcoxon rank sum test for difference in the median AUROC for our machine learning model using EHR data alone, and our model that utilized EHR, survey, and genetic data. This increase was significant (p<0.001) in a Wilcoxon test with a test statistic of 93.0. The addition of survey and genetic data in our XGBoost model also led to an increase in the observed mean accuracy (95% CI) from 0.480 (0.468, 0.493) to 0.521 (0.504, 0.537) (Figures 2,4). Precision also increased from 0.056 (0.055, 0.057) to 0.061 (0.059, 0.063). F1 showed a similar increase from 0.104 (0.102, 0.107) to 0.113 (0.109, 0.117). Specificity also increased significantly from 0.468 (0.455, 0.481) to 0.510 (0.492, 0.527). All of these increases were observed when recall was held constant at 0.800 (Figure 2). These findings show that the addition of survey and genetic data leads to an overall increase in the quality of the model’s prediction.

Identifying participants with long COVID using the TabPFN framework with EHR, survey, and genetic features achieved a mean AUROC (95% CI) of 0.738 (0.732, 0.744). The mean (95% CI) accuracy, precision, F1, and specificity were 0.494 (0.480, 0.508), 0.057 (0.056, 0.059), 0.107 (0.105, 0.110), and 0.482 (0.468, 0.497), respectively. When trained using only EHR features, TabPFN achieved a mean AUROC (95% CI) of 0.729 (0.722, 0.735). The mean (95% CI) accuracy, precision, F1, and specificity were 0.476 (0.465, 0.488), 0.055 (0.054, 0.057), 0.104 (0.102, 0.106), and 0.464 (0.452, 0.476), respectively. The difference in mean AUROC between TabPFN with all features and the model with only EHR features was statistically significant (p < .001).

**Figure 3:**
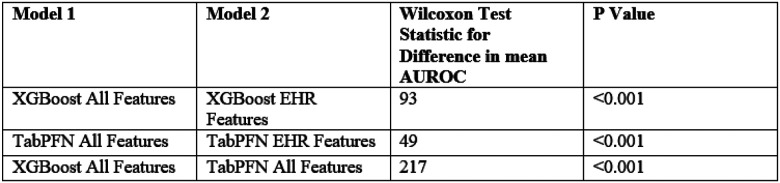
Wilcoxon Test Results for Difference in Mean AUROC

The XGBoost model outperformed the TabPFN model on AUROC score when using survey, genetic, and EHR data. This difference was significant in a Wilcoxon test for difference in median AUROC with a test statistic of 262 (Figure 3).

### Contribution of Individual Features

We implemented SHAP to explain and interpret the use of features in our machine learning models.^16^The utilization of shapely (SHAP) values in this way has been useful in previous research to find the relative contribution of aggregated features for model predictions.^17^

**Figure 4:**
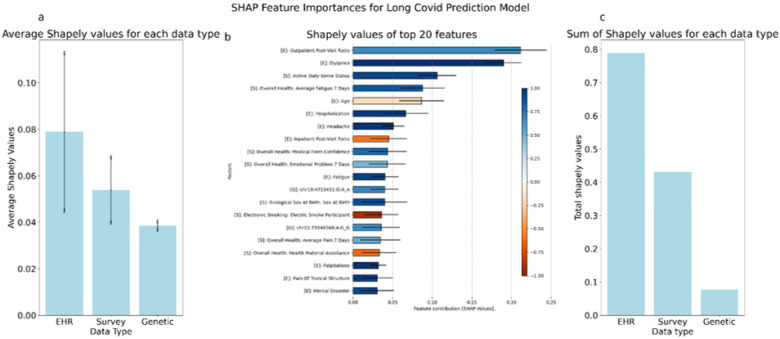
Feature Importance of Top 20 features by Data Type Using Shapely Values

To identify the most important predictive features in our algorithm, we calculated the SHAP values for each feature using the full XGBoost model and ranked the contributions of the top 20 features to the model’s performance. Out of the top 20 features, 10 were EHR data features, 8 were survey data features, and 2 were genetic data features. The five highest contributing features were the proportion of outpatient visits per month a participant had after their COVID19 diagnosis, a diagnosis for dyspnea or shortness of breath, military active-duty service status reported in a survey, self-reported fatigue from survey data, and a participant’s age. The full list of features and the directionality of an association with long COVID is shown in Figure 4.

### Contribution of Data Sources to Predicting Long COVID

To estimate the relative contribution of each data modality— EHR, survey, and genetic data — to the overall predictive performance, we trained separate XGBoost models using each data modality and combined their predicted probabilities using a weighted average. We used a constrained optimization via the Sequential Least Squares Programming algorithm to minimize the log loss on the validation set such that all weights were constrained to be non-negative and summed to one. This method yielded average weights of 78.4% for EHR data, 21.5% for survey data, and 0.01% for genetic data across 50 cross-validation folds (Figure 4).

This identified EHR-derived features as the primary drivers of predictive performance for long COVID in All of US, -with participant survey data also having modest contribution to the overall prediction. Although genetic data did not have a large overall performance, individual genetic factors were represented in the top twenty model features by contribution (Figure 4).

## DISCUSSION

Our results demonstrate that incorporating survey and genetic data sources modestly but significantly improves the overall accuracy and AUROC of a long COVID prediction model. The inclusion of different data modalities in AoU led to a measurable gain in model accuracy and AUROC compared to EHR-only models. This observation is consistent for both TabPFN and XGBoost based models. The increase of the AUROC of the models with the inclusion of genetic and survey data shows that previously discovered associations between social determinants of health and genetic factors can also aid in algorithmic prediction of long COVID when paired with EHR data. Given the consistency across multiple model frameworks, this method shows promise in other machine learning models based on EHR data and could lead to a similar increase in the AUROC.

Notably, feature importance analyses indicated that 8 of the 20 most influential features correspond to survey-derived features—capturing behavioral, social, and self-reported health information, whereas the predictive value of genetic features, though present, was smaller. Notable survey features included active-duty service status, self-reported fatigue, medical form confidence, and self-reported mental health problems within 7 days of the survey all of which were associated with a higher predicted risk of long COVID.

A meta-analysis of post COVID19 conditions publications in 2024 shows a noticeable gap in research on post COVID19 conditions within military populations, with only a few mentions in literature.^18^ Our results show that further research in this area may be beneficial to determine mechanisms for post COVID related symptoms in US military personal and recent veterans. Unfortunately, because the association has not been well understood, it is hard to tell whether there is a causal relationship between military involvement and long COVID, or if the differences could be attributed to clinical practices in military and veteran care sites for diagnosing long COVID.

Fatigue, has been a well-defined symptom of long COVID recognized by the CDC.^19^ Our findings of the importance of survey reported fatigue in our machine learning model for predicting long COVID confirms the symptom’s occurrence, and shows the importance of using additional information outside of the medical record to determine the symptom of fatigue in an individual. The CDC also recognizes brain fog, depression and anxiety as symptoms of long COVID19.^19^ The relationship between these variables and factors such as medical form confidence and self-reported mental health problems is unclear and shows room for further investigation into biological mechanisms between these variables. Because self-reported fatigue had a higher contribution to the model than fatigue diagnosis found in the EHR system, the differences in how fatigue is classified may be important in the prediction of long COVID.

Given the substantial burden of long COVID and the heterogeneity in its diagnosis and management across healthcare systems, our findings underscore the potential utility of machine learning algorithms as clinical decision-support tools.^14^ In particular, survey-based features such as self-reported fatigue and active-duty military status emerged as strong indicators of risk. These results suggest that broader integration of structured participant-reported data into clinical workflows—such as through brief intake surveys—could enhance early identification of individuals at risk for long-term COVID-19 complications.

Although TabPFN was recently reported to outperform tree-based models, such as XGBoost, when evaluated over more than 50 benchmark tabular datasets containing up to 10,000 training samples^15^, we did not observe the same advantage in the long COVID prediction task. We posit multiple non-mutually exclusive reasons for this phenomenon. First, the data for TabPFN pre-training relies upon dense, continuous, low-cardinality features, whereas long-COVID features are dominated by high-sparsity features, which is precisely where gradient-boosted trees shine but sits far outside TabPFN’s training distribution. Second, the outcome prevalence for long COVID is highly imbalanced (3.78%), and TabPFN currently lacks built-in class-weighting or focal-loss options; as a result, it may overfit the majority class, while XGBoost handles imbalance effectively through tailored objective functions and scale-pos-weight tuning. Third, extensive hyper-parameter optimization pipelines already exist for tree-based ensembles, whereas TabPFN exposes only a handful of tunable parameters, limiting our ability to adapt it to complex, domain-specific covariate interactions.

This study also has several limitations. Our original models were trained in part to make use of the features from the N3C teams’ machine learning model. Because of this, the biases and limitations present in the N3C model likely carry over to our predictive method. Given the uniquely rich and comprehensive data available in *All of Us* compared to other EHR biobanks, our algorithm based on AoU data has not been validated with any external datasets. Validation of our model represents a particular challenge given our use of *All of Us’* survey data; however, our findings may also encourage the collection of similar survey data on a broader scale. Furthermore, we did not assess the temporal relationship between when survey data was collected compared to when participants experienced their index COVID-19 infection, therefore whether the survey responses reflected participants’ pre-morbid or post-COVID state remains to be assessed. Because our data depended on participants with a COVID-19 diagnosis date in their EHR history, our algorithm cannot be used to predict long covid risk for undiagnosed COVID-19 cases, or COVID-19 cases without a clear diagnosis date. We only used preidentified COVID-19 related genetic variants instead of whole genome sequencing data. As such, the genetic contribution is limited known genetic indicators of COVID-19 hospitalization and contraction. AoU data contains its own limitations as well, including an age restriction of at least 18 years, different observational periods for each participant, and occasional missing data points. The combined model weighting method also has limitations. By combining predictions from different data sources through a linear weighted average, they may miss nonlinear relationships or interactions between data sources. Additionally, because models from each data source are trained independently, the combined model does not capture potential feature-level interactions.

Despite these constraints, our findings offer a promising path forward for precision risk stratification in long COVID. The approach we outline—enhancing existing models by incorporating new, multi-scale data types—provides a generalizable framework that can be applied to other clinical prediction tasks as additional data sources become available. Future work should focus on validating these models in external and prospective cohorts, expanding the range of genetic markers analyzed, and exploring how the integration of both genetic and participant-reported data can be streamlined into clinical practice.

## METHODS

### Data Source and Cohort

Data for this study was derived from AOU data^5^ version 8 in the production environment,^9^ which consist of electronic health records (EHRs), survey-based information, and genotyping data of non-deceased adults (18 years or older) living in the United States. EHR data encompasses health-care visit details, medical conditions, and prescription drug orders for each participant.

Our cohort inclusion criteria largely paralleled previous work from an original N3C study on predicting long COVID.^3^ Specifically, cohort members were required to have either 1) an International Classification of Diseases, Tenth Revision, Clinical Modification (ICD-10-CM) COVID-19 diagnosis code (i.e., U07.1) from an inpatient or emergency health-care visit, or 2) a positive SARS-CoV-2 PCR or antigen test.

We required AoU participants to have EHR data available in AoU both before and after the diagnosis date or positive test result. We excluded participants with less than 90 days between the positive COVID-19 test or diagnosis date and the end of the study (October 1, 2023) to ensure sufficient follow-up to assess long COVID.^3^ To accommodate the existing EHR data model, we gathered EHR data from AoU participants. We only gathered variables that were used in the pre-existing models and made only the exact same transformations based on the previous study’s data cleaning protocol.^6^ We used EHR data in the AoU production environment, which follows the common OMOP standard data model. The only additional inclusion criterion was that participants have valid survey and genetic data available in AOU. The classification of valid data for each is described below.

From the genetic data, we used discrete variables showing the minor allele counts (0, 1, and 2) for each genetic variant. We extracted 25 genetic variants from AoU whole genome sequencing (WGS) data version 7 **(Supplementary Table 1)**. These variants were effect alleles identified by the COVID-19 Genetic Consortium and were significantly associated to COVID-19 infection or hospitalization due to COVID-19.^20^ Participants with genetic data were included only if they had non-null values for the genetic expression of each of our pre-specified 25 genetic variants **(Supplementary Table 1)**.

From the health survey information available in AoU, we selected 32 questions which gave information that was distinctively different from the data available through our EHR data **(Supplementary Table 2)**. These questionnaire outputs were transformed into scalar variables for model testing and training. Participants considered to have valid survey data must have completed the AoU core survey questionnaire (the basics, lifestyle, and overall health) within the time span of their EHR history in AoU. Further, participants who skipped 10% or more of the questions used for our algorithms were excluded.

Data cleaning, feature engineering, and model training were completed using Python Jupyter Notebooks in the AoU Researcher Workbench platform and using cloud computing.

### Training, Validation, and Testing Sets

We randomly allocated 20% of the original dataset for model testing and used the remaining 80% of for model training and validation. This random split was independently repeated 10 times to validate the results across multiple random assortments. For each split, we conducted a 5-fold cross-validation on the 80% portion. Within each fold, a model was trained on the training set and hyperparameters were selected using a grid search based on the performance on the validation set. The reserved testing set was used to evaluate overall model performance and derive feature importance. (Figure 1).

### Model Design and Machine Learning Infrastructure Selection

We implemented the N3C model to determine baseline performance for all data sets. We then trained two models independently: (1) genetic data alone, and (2) survey data. We then created XGBoost and TabPFN models for comparison (Figure 1).

Additionally, we applied sigmoid regression on the validation set to calibrate each model using the CalibratedClassifierCV function from the scikit-learn python package. Calibration is important for preserving the integrity of the predictive probability given by our classification model outputs. A calibrated model has more interpretive value in disease classification.

### Cross Validation

We created a set of parameters for training models and validated our results using an original cross validation method. We trained each model separately using a 5-fold cross validation method **(Supplementary Table 3)**. We assessed AUROC, positive predictive values, recall, specificity, and sensitivity for each iteration. We used a Wilcoxon Test Statistic for difference in model performance to assess the significance of difference between the AUROC (Figure 3).

### Assessing Individual Predictive Features

By calculating Shapely values^16^, we determined the most important features in the final predictive method based on Shapley values and the relative direction for each new feature. To do so, we calculated the mean SHAP values and the standard derivation and then ranked the features based on their SHAP values to identify the 20 most influential features. The resulting proportional feature contributions for each model were then combined by multiplying each model’s set of Shapely values, by the model’s relative contribution to the overall predictive method.

## Supplementary Files

This is a list of supplementary files associated with this preprint. Click to download.

- SupplementaryTablesAndLegend.docx

## Figures and Tables

**Table 1.** Demographic Distribution of All of Us Study Cohort

Demographic	Value	Cases	Controls	Total Population
Total Cohort		>=650	>=16,600	>=17,200
Sex at Birth	Male	178	5408	5586
	Female	471	11051	11522
	Unknown	<20	145	>=145
Gender	Woman	468	10992	11460
	Man	175	5364	5539
	Other	<20	105	>=105
	Unknown	<20	143	>=143
Ethnicity and Race				
	Hispanic	95	2937	3032
	White	348	8590	8938
	Black	126	3096	3222
	Asian	<20	327	>=327
	American Indian or Alaskan Native	<20	88	>=88
	Middle Eastern and North African	<20	85	>=85
	Native Hawaiian or Pasific Islander	<20	<20	<40
	Generalized Multiple Populations	46	1055	1101
	Unknown	27	420	447
Age	Mean	58.5	57.7	57.7
	Median	60	59	59
	Minimum	23	20	20
	Q1	49	45	45
	Q3	69	70	70
	Maximum	99	105	105

**Table 2.** XGBoost predictive model performance with 95% confidence interval for predicting long COVID using different data sources.

Model	AUROC	Accuracy	Precision	F1	Recall	Specificity
All Data Combined	0.748 (0.741, 0.755)	0.521 (0.504, 0.537)	0.061 (0.059, 0.063)	0.113 (0.109, 0.117)	0.800 (0.800, 0.800)	0.510 (0.492, 0.527)
Survey Data	0.630 (0.625, 0.636)	0.046 (0.045, 0.046)	0.800 (0.800, 0.800)	0.086 (0.085, 0.088)	0.800 (0.800, 0.800)	0.343 (0.331, 0.355)
Genetic Data	0.527 (0.519, 0.534)	0.191 (0.167, 0.216)	0.038 (0.038, 0.039)	0.073 (0.072, 0.074)	0.842 (0.821, 0.863)	0.166 (0.140, 0.192)
EHR data	0.736 (0.730, 0.741)	0.480 (0.468, 0.493)	0.056 (0.055, 0.057)	0.104 (0.102, 0.107)	0.800 (0.799, 0.801)	0.468 (0.455, 0.481)

EHR=electronic health record; AUROC=area under the receiver operating characteristics curve; PPV=positive predictive value

## Data Availability

The All of Us dataset can be accessed through the Researcher Workbench by following the detailed data application process outlined at https://www.researchallofus.org.

## Code Availability

The source code associated with this study will be publicly available at: https://github.com/The-Wei-Lab/AoU-COVID-Machine-Learning. At the time of this journal’s publication. Prior to publication, the code is available to reviewers through All of Us Researcher Workbench on request.
